# Generation of a novel HEK293 luciferase reporter cell line by CRISPR/Cas9-mediated site-specific integration in the genome to explore the transcriptional regulation of the PGRN gene

**DOI:** 10.1080/21655979.2019.1607126

**Published:** 2019-04-26

**Authors:** Yanqing Li, Sai Li, Yan Li, Haibin Xia, Qinwen Mao

**Affiliations:** a Laboratory of Gene Therapy, Department of Biochemistry, College of Life Sciences, Shaanxi Normal University, Xi’an, Shaanxi, P.R. China; b Department of Pathology, Northwestern University Feinberg School of Medicine, Chicago, IL, USA

**Keywords:** CRISPR/Cas9, PGRN, knock-in, luciferase, transcriptional regulation, HEK293

## Abstract

Progranulin has multiple functions in several physiological and pathological processes, including embryonic development, wound repair, tumorigenesis, inflammation and neurodegeneration. To investigate the transcriptional regulation of the PGRN gene, a luciferase knock-in reporter system was established in HEK293 cells by integrating luciferase gene in the genome controlled by the endogenous PGRN promoter using CRISPR/Cas9. PCR results demonstrated the site-specific integration of the exogenous luciferase gene into the genome. To validate the novel luciferase knock-in system, a CRISPR/Cas9 transcription activation/repression system for the PGRN gene was constructed and applied to the knock-in system. In addition, phorbol ester (phorbol 12-myristate, 13-acetate), previously reported as activating the expression of PGRN, was applied to the system. The results indicated that luciferase activity was directly correlated with the activity of the PGRN endogenous promoter. This novel system will be a useful tool for investigating the transcriptional regulation of PGRN, and it has great potential in screening the drugs targeting PGRN.

## Introduction

Progranulin (PGRN) is a 68.5 kDa, cysteine-rich protein with a length of 593 aa, which is typically secreted in its 88 kDa form with high glycosylation [1,2]. Extracellular proteases such as elastase, neutrophil-derived protease, MMP-9, MMP-14 or ADAMTS7 can proteolytically cleave PGRN into paragranulin and seven domains which were named as granulin (Grn) G, F, B, A, C, D and E, respectively. Structurally, each Grn contains 10–12 highly conserved cysteine residues, none of which belongs to well-established growth factor families, but each having individual functions [3,4].

PGRN plays a role in embryonic development, tissue repair, tumorigenesis, inflammation and neurodegeneration [5,6]. High levels of PGRN expression have been associated with several human cancers and are believed to contribute to tumorigenesis in breast cancer, non-small cell lung carcinoma, invasive ovarian carcinoma, glioblastoma, and multiple myeloma [7–11]. Serum PGRN levels are increased in obese and type 2 diabetic patients [12]. The decreased expression of PGRN has been reported to involve in frontotemporal lobar degeneration [13]. Hence, to investigate the regulation of PGRN gene transcription is very important for exploring PGRN gene function and screening the drugs targeting PGRN. However, to establish a sensitive and efficient method to monitor the transcription of PGRN gene is challengeable.

Methods such as real-time PCR, Western blot and reporter gene expression controlled by an exogenous promoter have been widely used to investigate transcriptional regulation. However, compared with these conventional methods, analyzing target gene transcription by luciferase reporter gene expression under the control of an endogenous promoter is more precise and sensitive, especially for high-throughput screening (HST).

Targeted genome editing by homologous recombination is widely used to study gene function. Programmable nucleases such as zinc finger nucleases (ZFNs) and transcription activator-like effector nucleases (TALENs) have been developed for targeted genome engineering [14,15]. Programmable nucleases generate site-specific DNA double-strand breaks (DSBs) in the genome, which induce targeted genome modifications through cellular repair mechanisms. Recently, the clustered regularly interspaced short palindromic repeat (CRISPR)/Cas9 (CRISPR-associated) system responsible for adaptive immunity in bacteria has been repurposed for genome engineering and provides an advanced platform for gene functional studies in biomedical research [16–19]. To modify the genome precisely, DSBs need to be introduced at a preselected chromosomal locus [20]. In this study, to explore PGRN transcriptional regulation and to promote functional research into the PGRN gene, we used CRISPR/Cas9 to knock-in a luciferase reporter gene in the genome under the control of the endogenous PGRN promoter in HEK293 cells to monitor the activity of the promoter of the PGRN gene.

## Materials and methods

### Cell line

HEK293 (human embryonic kidney) cells were purchased from American Type Culture Collection (ATCC, Manassas, VA, USA). The cells were cultured in Dulbecco’s modified Eagle’s medium (Gibco, Grand Island, NY, USA) supplemented with 10% fetal bovine serum (Gibco, Grand Island, NY), 1% penicillin-streptomycin and 1% L-glutamate. The cells were cultured in a humidified chamber with 5% CO_2_ at 37°C.

### sgRNA design

For site-specific integration of the luciferase reporter in the genome controlled by the endogenous promoter of PGRN, four sgRNAs were designed using the CRISPR scan website (http://www.crisprscan.org/). sgRNAs targeting different areas located right after the stop codon of the human PGRN gene were selected (Table 1 in Supplementary Material). To construct the CRISPR/Cas9-based transcription activation/repression system of the PGRN promoter, seven sgRNAs targeting PGRN promoter areas were selected (Table 2 in Supplementary Material).

### Donor vector construction

A 996 bp homologous arm located upstream of the transcriptional termination site of PGRN and a 781 bp homologous arm located downstream of the translation termination site were amplified by PCR. The construction process of pUC19/PGRN donor was similar to that of pUC19/Sox2 donor, as previously reported [21]. pUC19/PGRN donor vector included positive and negative screening elements. A CMV-eGFP-T2A-Neomycin-SV40pA expression cassette was used as the positive selection and a PGK-TK-T2A-mCherry-SV40pA expression cassette was used as the negative selection. All primers for amplifying the upstream and downstream homologous arms are listed in Table 2 in Supplementary Material.

### Construction of CRISPR/Cas9 transcription activation/repression system

To validate luciferase knock-in cell line, CRISPR/Cas9-based transcription activation system were constructed as previously reported [21,22], consisting of MS2-p65-HSF1, Cas9-VP64 and sgRNA1.0-X (where X stands for 1–7) or sgRNA2.0-X (where X stands for 1–7) targeting PGRN promoter region. And CRISPR/Cas9-based transcription repression system, consisting of Cas9-KRAB, and sgRNA1.0-X (where X stands for 1–7) or sgRNA2.0-X targeting PGRN promoter region, was generated according to a previously reported method [23]. The sgRNA expression vectors pU6-sgRNA1.0 and pU6-sgRNA2.0 were constructed according to methods described previously [22]. pU6-PGRN sgRNA1.0 and pU6-PGRN sgRNA2.0 containing sgRNAs targeting mouse glucuronidase gene were used as control vectors.

To assay CRISPR/Cas9-based transcription activation/repression system, an exogenous PGRN promoter sequence was amplified by PCR and then ligated to pGL3 basic vector (Promega, Madison, WI). The obtained plasmid was named pGL3/PGRN promoter (PGRNp). The primers used for PCR amplification were shown in Table 2 in Supplementary Material.

### Establishment of HEK293-PGRN-T2A-LUCIFERASE-KI cell line

To establish a CRISPR/Cas9-mediated luciferase knock-in reporter system under the control of the endogenous PGRN gene promoter in HEK 293 cells (HEK293-PGRN-Luciferase knock-in (KI) cell line), 4 µg of targeting vector of pUC19/CMV-Cas9-PGRN sgRNA2 and 8 μg of pUC19/PGRN donor vector were co-transfected into HEK293 cells plated in a 60 mm dish at an efficiency of ∼80% using Lipofectamine 2000 Transfection Reagent (Invitrogen, Carlsbad, CA). The cells grew stably by G418 (200 µg/ml) and gancivlovir (1.0 mg/ml) screening, then HEK293-PGRN-T2A-Luciferase-KI cell line was obtained by further cloning by limited dilution assay.

### Analysis of site-specific integration of the exogenous gene in hek293-pgrn-t2a-luciferase-ki cell line

Genomic DNA was isolated from HEK293-PGRN-T2A-Luciferase-KI cells using the TIANamp Blood DNA Kit (Tiangen, Beijing, China). PGRN gene-targeted integration was detected by PCR using PrimeSTAR HS DNA polymerase (Takara, Dalian, China). PCR products were purified and then cloned into pGEM-T Easy Vector (Promega, Madison, WI, USA) for sequencing. The primers used for PCR were shown in Table 2 in Supplementary Material.

### T7E1 assay

To screen the biological activity of sgRNAs targeting different areas located right after the stop codon of the human PGRN gene, pU6/PGRN sgRNA1.0-X (where X stands for sgRNA1–4) was constructed by separately cloning four sgRNA oligonucleotide duplexes into the *Bsa*I-digested pU6-sgRNA1.0. Then, 3 µg of pU6/PGRN sgRNA1.0-X (where X stands for sgRNA1–4) expressing vector/control vector and 6 µg of pCMV-Cas9 were co-transfected into HEK293 cells plated in a 60 mm dish (2 × 10^6^ cells per dish). Genomic DNA was extracted from experimental and control cells using the TIANamp Blood DNA Kit (Tiangen, Beijing, China). An 863 bp fragment flanking the binding sites of CRISPR/Cas9 was amplified by nest PCR using the detection primers. The PCR product was then purified with an AxyPrep DNA Gel Extraction Kit (Axygen, Hangzhou, China). For the T7E1 assay, the purified product was denatured and reannealed, then digested with T7E1 (New England Biolabs, Ipswich, MA). The digested product was then separated by DNA gel electrophoresis and photographed. The primers used for the T7E1 assay were listed in Table 2 in Supplementary Material.

### Validation of HEK293-PGRN-T2A-LUCIFERASE-KI cell line

To detect whether the luciferase expression was correlated with the endogenous PGRN promoter, we first screened sgRNA for its effectiveness in regulating hPGRN gene expression based on the CRISPR/Cas9 transcription activation/repression system by co-transfecting 0.4 μg of pCas9-VP64, pMS2-p65-HSF1 and pU6-PGRN sgRNA2.0 expressing vector/control vector or 0.4 μg of pCas9-KRAB and pU6-PGRN sgRNA1.0 expressing vector/control into HEK293 cells with 0.4 μg pGL3/PGRNp into HEK293 cells plated in a 24-well plate (2 × 10^5^ cell per well). 48h post transfection, HEK293 cells were collected to analyze luciferase activity. Then, the sgRNAs with the highest transcription activation or repression activity were selected for the construction of the vector carrying multiple sgRNAs expressing cassettes. Next, 0.4 μg of pCas9-VP64, pMS2-p65-HSF1, and pU6-PGRN sgRNA2.0-X (where X stands for the sgRNA significantly upregulating the activity of the PGRN promoter)/control vector or 0.4 μg of pCas9-KRAB and pU6-PGRN sgRNA1.0-X (X stands for the sgRNA significantly downregulating the activity of the PGRN promoter)/control vector was co-transfected into HEK293-PGRN-T2A-Luciferase-KI cells or HEK293 cells. 72 h post transfection, HEK293-PGRN-T2A-Luciferase-KI and HEK293 cells were collected to analyze luciferase activity. HEK293 cells were collected to analyze the expression of endogenous PGRN by real-time PCR. The primers used for real-time PCR are listed in Table 2 in Supplementary Material.

To demonstrate the utility of HEK293-PGRN-T2A-Luciferase-KI cell, different doses (0, 0.2, 0.5, 1, 2.5 and 6.25 ng/ml) of PMA were added to HEK293-PGRN-T2A-Luciferase-KI cells plated in a 24-well plate (2 × 10^5^ cell per well). 48 h later, the cells were collected to analyze luciferase activity. HEK293 or HEK293-PGRN-T2A-Luciferase-KI cells were treated with effective dose of PMA (2.5 ng/ml) for 48 h, then the indicated cells were harvested to analyze the endogenous PGRN mRNA level, and the supernatants from the indicated cells were collected to analyze the level of PGRN protein using PGRN ELISA kit according to the Manufacturer’s instructions. The luciferase activity was assayed using a luciferase assay kit (Promega, Madison, WI).

Lastly, to further demonstrate the utility of the sensor system, we used the established cell line to screen small molecule chemical drugs that could affect the expression of PGRN from a library of 28 inflammation and lipid metabolism-related drugs from Selleck Chemicals. The drug information corresponding to the specific drug number can be found in the Selleck website (https://www.selleck.cn/). All drugs were dissolved in DMSO and the final working concentration was 10 µM. HEK293-PGRN-T2A-Luciferase-KI cells were plated in a 96-well plate (1 × 10^4^ cell per well). After 24 h, each drug was treated with three wells. Negative control (DMSO) group and blank control (DMEM medium) group were set. 48 h later, the cells were collected to analyze luciferase activity. Different concentration gradients (2.5, 5, 10, 20 µM) of S2349 (Rutaecarpine) were added to HEK293-PGRN-T2A-Luciferase-KI cells plated in a 96-well plate (1 × 10^4^ cell per well). 48 h later, the cells were collected to analyze luciferase activity. HEK293 were treated with effective dose of S2349 (10 µM) for 48 h, then the indicated cells were harvested to analyze the endogenous PGRN mRNA level.

### Statistical analysis

Statistical analyses were performed using GraphPad Prism version 6.0 software. Results are representative of three independent experiments. The comparison among the multi-group measurement data was performed using analysis of variance (one-way ANOVA). Student’s *t*-test was used to analyze the statistical signiﬁcance of two groups. Error bars show the means ± SD. *P < 0.05; **P < 0.01; ***P < 0.001; NS, not significant. Primer pairs for the seven sgRNAs targeting the PGRN promoter were synthesized by BGI (Beijing, China) and annealed at room temperature for 2 h to form sgRNA oligonucleotide duplexes. Then, each sgRNA oligonucleotide duplex was ligated with the *Bsa*I digested pU6-sgRNA2.0 or pU6-sgRNA1.0.

## Results

### Generation of the HEK293-PGRN-T2A-LUCIFERASE-KI cell line

**Figure 1.**
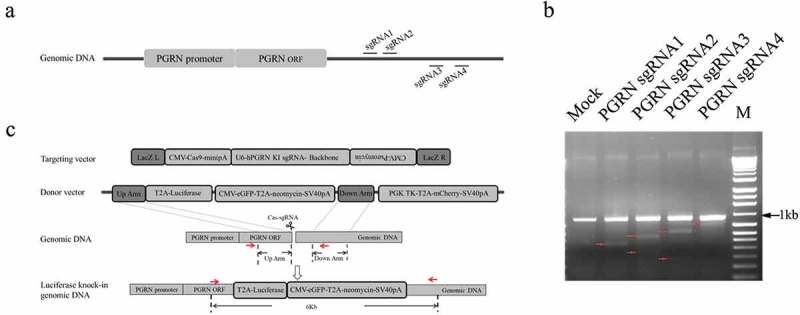
Schematic diagram of CRISPR/Cas9-mediated site-specific integration of exogenous gene in the genome. (a) An illustration of the target sites of the designed sgRNAs in the genome. (b) Analysis of the efficacy of designed sgRNAs by T7E1 assay. Red arrows indicate the expected positions of the DNA bands cleaved by T7E1. (c) Strategy of CRISPR/Cas9-mediated site-specific integration of the exogenous gene in the genome. Red arrows indicate the location of primers for genotyping the cell line.

CRISPR/Cas9 was used to knock-in a luciferase reporter gene in the genome under the control of the endogenous PGRN promoter. To achieve this goal, four sgRNAs targeting the region after the stop codon of the PGRN gene were selected, then the biological activity of these sgRNAs was analyzed by T7E1 assay. The results showed that sgRNA2 had the highest gene-editing activity among sgRNA1–4 (Figure 1a,b). A targeting vector that included Cas9 driven by the CMV promoter and sgRNA2 driven by the U6 promoter was constructed according to the method described previously [21] (Figure 1c). The donor vector generated in the study included a TK-T2A-mCherry expression cassette driven by the PGK promoter for negative screening purposes, a luciferase reporter gene inserted in the 3ʹterminal of the left homologous arm followed by an eGFP-T2A-neomycin expression cassette driven by CMV promoter for positive screening (Figure 1c).

**Figure 2.**
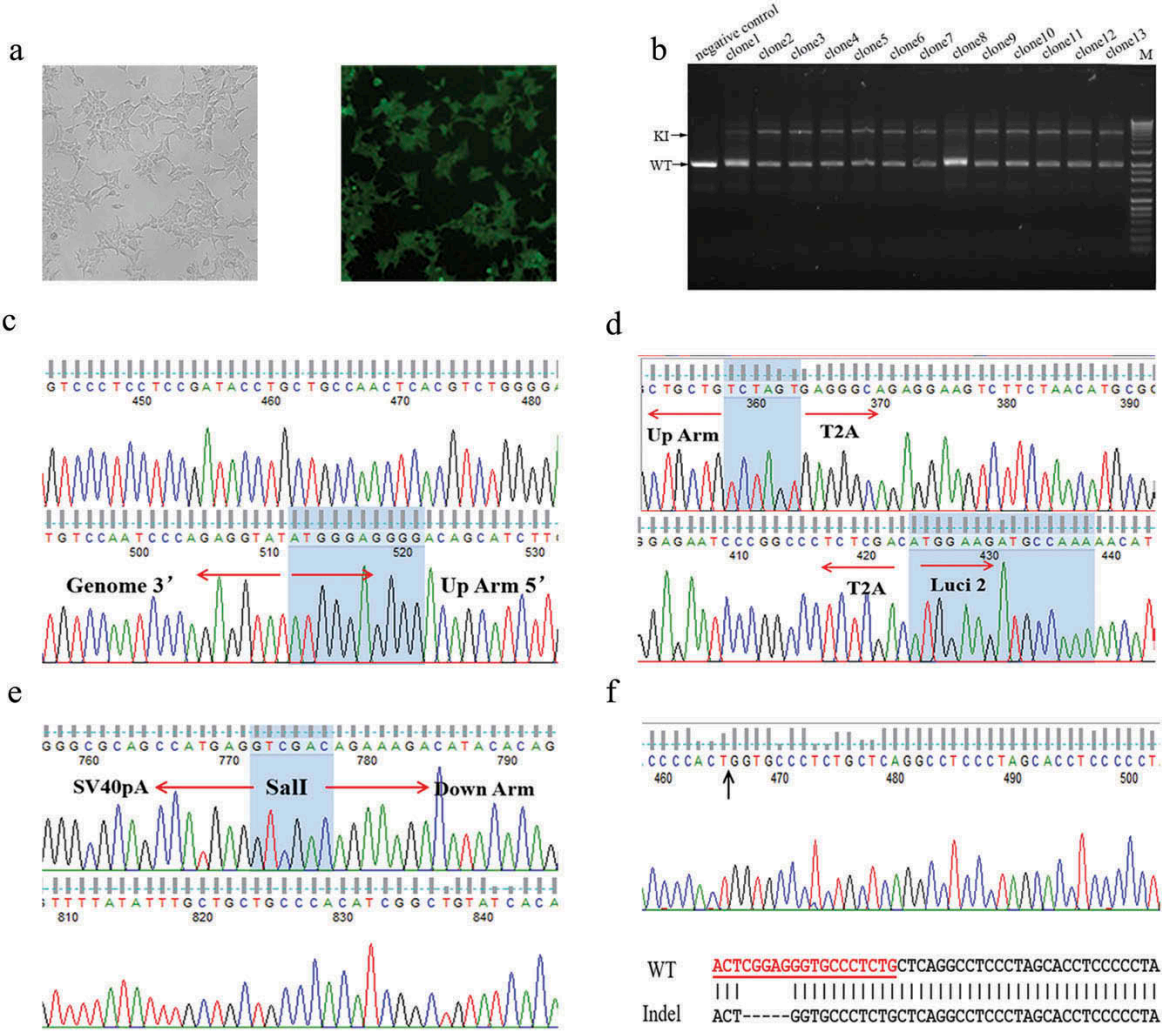
Establishment of the HEK293-PGRN-T2A-Luciferase-KI cell line. (a) The HEK293-PGRN-T2A-Luciferase-KI cell line with stable expression of eGFP and undetectable expression of mCherry. (b) Genotyping of the positive clones contained a lower band corresponding to the WT and an upper band corresponding to the targeted integration (TI). (c) Sequencing results of the 5ʹ and 3ʹ terminal sequences from the upper band of clone 4 in panel B. (d) Sequencing results of the up homologous arm from the upper band of clone 4 in panel B. (e) Sequencing results of the down homologous arm sequences from the upper band of clone 4 in panel B. (f) Sequencing results from the lower band of clone 4 in panel B. The lower band contained a 5 bp deletion.\

To establish a HEK293 cell line with a knock-in luciferase gene under the control of the endogenous PGRN gene (named HEK293-PGRN-T2A-Luciferase-KI), targeting vector and donor vector were co-transfected into HEK293 cells followed by positive and negative screening in the presence of G418 and Ganciclovir (GCV) for ~20 days. The cells showed eGFP expression with undetectable expression of mCherry, indicating the site-specific integration of the exogenous gene into the genome (Figure 2a). A total of 39 primary clones were isolated and examined by PCR. The results revealed a large fragment with a length of 6.0 kb in each clone, indicating a modified allele with the integration of the exogenous gene, and a smaller band with a size similar to that amplified from the wild-type (WT) HEK293 cells (Figure 2b). The two bands amplified from clone 4 were then used for further investigation. The large band was shown by sequencing to be a specific integration of the exogenous luciferase gene (Figure 2c–2e). The smaller band, interestingly, was shown to be a product of (Non-homologous end joining, NHEJ)that resulted in a 5-bp deletion (Figure 2f). The cell line derived from clone 4, named HEK293-PGRN-T2A-Luciferase-KI, was stored for subsequent experiments.

### Validation of the HEK293-PGRN-T2A-LUCIFERASE-KI cell line in vitro

**Figure 3.**
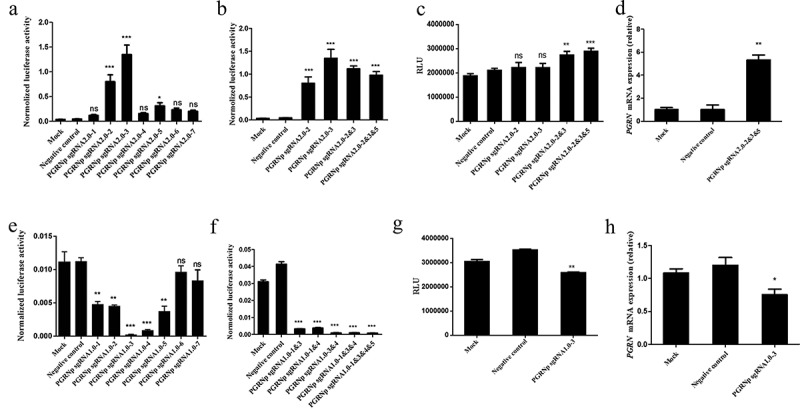
The regulatory effects of the CRISPR/Cas9-based transcription activation/repression system on the endogenous promoter of the PGRN gene. Comparison of the CRISPR/Cas9-based transcription activation effect carrying different single sgRNAs (a) or multiple sgRNAs (b) on exogenous PGRN promoter activity in HEK293 cells. The effect of the dCas9-VP64 transcription activation system carrying different combination of 2.0-sgRNAs (c) or 2.0-sgRNA2&3&5 (d) on the endogenous PGRN promoter activity by detecting luciferase activity in the HEK293-PGRN-T2A-Luciferase-KI cell line or PGRN mRNA expression in HEK293 cells. The CRISPR/Cas9-based transcription repression effect carrying different single sgRNAs (e) or multiple sgRNAs (f) on exogenous PGRN promoter activity in HEK293 cells. The dCas9-KRAB transcription repression system carrying sgRNA1.0–3 on the endogenous PGRN promoter activity by detecting luciferase activity in the HEK293-PGRN-T2A-Luciferase-KI cell line (g) and the corresponding endogenous PGRN mRNA level by real-time PCR in the HEK293 cell line (h). RLU represented Relative light unit. Data are represented as means ± SD (*n* = 3; *P < 0.05；**P < 0.01; ***P < 0.001; ns, not significant; one-way ANOVA.

To investigate whether the expression of the luciferase knock-in reporter gene was under the control of the endogenous PGRN promoter, a CRISPR/Cas9-based transcription activation system targeting the PGRN promoter was generated according to a previously reported method [22]. Then, the biological activities of seven sgRNAs were assayed by co-transfecting pGL3/PGRNp and the plasmids for the CRISPR/Cas9-based transcription activation system into HEK293 cells (Figure 3a). The sgRNAs with high biological activity were selected for the construction of pU6-PGRN sgRNA2.0–2&3 or pU6-PGRN sgRNA2.0–2&3&5. These constructs were analyzed in HEK293 cells using a similar strategy as above. The results indicated that the transcriptional activity of the exogenous PGRN promoter was enhanced significantly, with an increased number of sgRNA expression cassettes in the context of the transcription activation system (Figure 3b). Next, the transcription activation system containing pU6-PGRN sgRNA2.0–2, pU6-PGRN sgRNA2.0–3, pU6-PGRN sgRNA2.0–2&3 or pU6-PGRN sgRNA2.0–2&3&5 was applied to the HEK293-PGRN-T2A-Luciferase-KI cell line. Compared with the negative control and mock, the luciferase activity assay showed an overall two-fold increase in the HEK293-PGRN-T2A-Luciferase-KI cell line treated with the transcription activation system. Interestingly, the cells treated with the transcription activation system containing multiple sgRNAs showed relatively high luciferase activity (Figure 3c). The real-time PCR results of endogenous PGRN mRNA expression levels in the HEK293 cell line treated with the CRISPR/Cas9 transcription activation system displayed a similar pattern as the results of luciferase activity from HEK293-PGRN-T2A-Luciferase-KI cell line treated with the transcription activation system (Figure 3d).

Using a similar strategy, we tested the CRISPR/Cas9-based transcription repression system containing single sgRNAs or multiple sgRNAs. PGRNp-sgRNA1.0–3 and PGRNp-sgRNA1.0–1&3&4&5 with higher activity for inhibiting exogenous PGRN promoter activity (Figure 3e,f) were selected to construct the CRISPR/Cas9-based transcription repression system. This system was tested in the HEK293-PGRN-T2A-Luciferase-KI cell line and in HEK293 cells. The result of the luciferase assay indicated that the CRISPR/Cas9-based transcription repression system containing PGRNp-sgRNA1.0–3 reduced luciferase activity compared with the negative control and mock (Figure 3g). Similar results were obtained with the assay of PGRN mRNA expression in HEK293 cells (Figure 3h).

**Figure 4.**
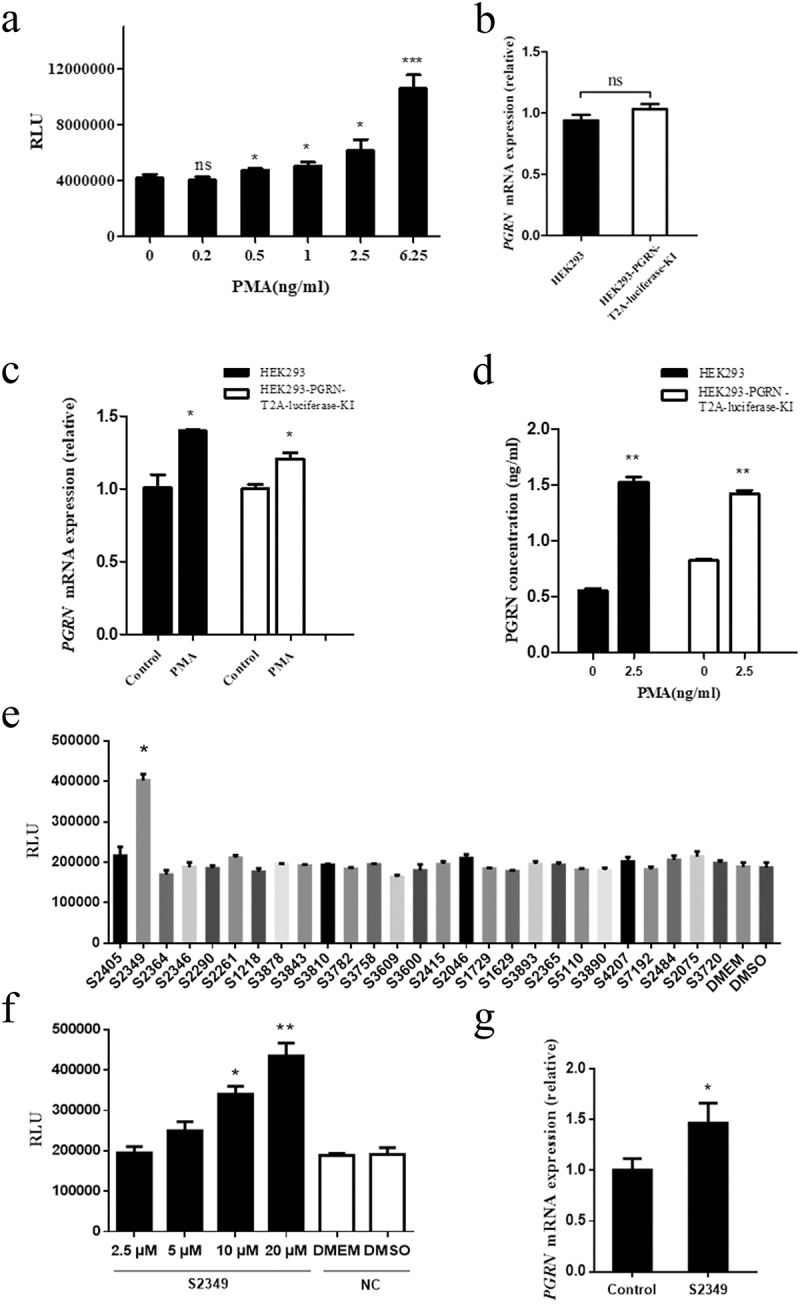
The effect of small molecule chemical drugs on PGRN transcription regulation. (a) Different doses of PMA were added to HEK293-PGRN-T2A-Luciferase-KI cell line. The luciferase activity was detected 48 h post-treatment with PMA. (b) The endogenous PGRN mRNA level was assayed in the HEK293 and HEK293-PGRN-T2A-Luciferase-KI cell lines in the presence of PMA by real-time PCR. The effect of PMA (2.5 ng/ml) on the endogenous PGRN mRNA level (c) and the PGRN protein level from supernatant (d) were analyzed in the HEK293 and the HEK293-PGRN-T2A-Luciferase-KI cell lines. (e) A library of 28 inflammation and lipid metabolism related small molecules were screened in HEK293-PGRN-T2A-Luciferase-KI cell line. (f) Different doses (2.5, 5, 10, 20 µM) of S2349 were added to HEK293-PGRN-T2A-Luciferase-KI cell line. The luciferase activity was detected 48 h post-treatment with S2349. (g) The endogenous PGRN mRNA level was assayed in the HEK293 treated with S2349 (10 µM) by real-time PCR. NC, negative control; ns, no significant. Statistically significant differences were indicated: *P < 0.05; **P < 0.01; ***P < 0.001; Student’s *t*-test.

Phorbol ester [phorbol 12-myristate,13-acetate (PMA)], previously reported to regulate PGRN expression [24], was also applied to the HEK293-PGRN-T2A-Luciferase-KI cell line. As shown in Figure 4a, luciferase activity was increased in cells treated with PMA compared with the negative control treated with only culture medium, suggesting that PMA treatment upregulated the transcriptional activity of PGRN in HEK293-PGRN-T2A-Luciferase-KI cells. The RT-PCR analysis indicated that endogenous PGRN mRNA expression level in the HEK293 cell line had no difference with that of the HEK293-PGRN-T2A-Luciferase-KI cell line (Figure 4b), suggesting that the reporter cell line was reliable and faithful in reflecting the transcriptional level of the PGRN gene. After treatment with PMA, the PGRN mRNA level was significantly improved both in HEK293 cells and the reporter cell line (Figure 4c). The PGRN protein levels in HEK293 cells and the reporter cell line treated with PMA were also significantly increased compared with the negative control (Figure 4d), which was consistent with the luciferase activity in the HEK293-PGRN-T2A-Luciferase-KI cell line treated with PMA. PGRN was a protein related to inflammation and lipid metabolism, which was involved in their molecular mechanism [25,26]. To further demonstrate the utility of the sensor system, a library of inflammation and lipid metabolism related small molecule chemical drugs was used to screen the drugs that could affect the expression of PGRN gene. We screened 28 small molecule drugs and found that S2349 (Rutaecarpine) can significantly promote luciferase activity compared with the negative control treated with DMSO (Figure 4e), and luciferase activity was improved with the increasing of the dose of S2349 (Figure 4f). RT-PCR result also showed that S2349 could promote the expression of PGRN mRNA (Figure 4g).The above evidence suggested that the expression of the luciferase knock-in reporter gene was directly related to the endogenous PGRN promoter and was a faithful reporter of the endogenous PGRN promoter.

## Discussion

PGRN, as a multifunctional regulatory protein, promotes mitosis, survival, and migration in many cell types. It stimulates growth factor-related signaling pathways such as the phosphorylation of shc, p44/42 mitogen-activated protein kinase, phosphatidylinositol 3-kinase, protein kinase B/AKT and the p70S6 kinase [27,28], and promotes the development and progression of many tumors. PGRN is also involved in many physiological processes such as wound repair [5], embryo development [6], blastocyst hatching [29] and male-speciﬁc differentiation of the neonatal hypothalamus [30,31]. Due to the important roles of PGRN in so many physiological and pathological processes, a sensitive, reliable method is needed for exploring the transcriptional regulation of PGRN expression. To achieve this goal, we established the HEK293-PGRN-T2A-Luciferase-KI cell line with luciferase expression under the control of the endogenous PGRN promoter using CRISPR/Cas9.

In general, gene expression can be analyzed using real-time PCR or Western blot; however, these methods are time-consuming, not sensitive and prone to variation. More importantly, they are not suitable for HST assay. The HEK293-PGRN-T2A-Luciferase-KI cell line created in this study, with luciferase reporter expression under the control of the PGRN endogenous promoter, can be used to sensitively, easily and quickly evaluate PGRN expression via HST assay.

To validate the HEK293-PGRN-T2A-Luciferase-KI cell line, CRISPR/Cas9-based transcription activation and repression systems were generated according to previously reported methods [22,23]. Compared with the exogenous PGRN promoter, the CRISPR/Cas9-based transcription activation/repression systems were shown to be less effective on endogenous promoter activity both in HEK293-PGRN-T2A-Luciferase-KI cells and HEK293 cells. This result may reflect an epigenetic difference between the exogenous and endogenous PGRN promoters. In addition, the chemical agent PMA was used to test the reporter cell line and showed significant regulatory activity on PGRN expression. To further demonstrate the utility of the sensor system, we used the established cell line to screen inflammation and lipid metabolism related small molecule chemical drugs that could affect the expression of PGRN gene. S2349 (Rutaecarpine) was a kind of small molecule drug screened from the drug library that can promote the expression of PGRN. Rutaecarpine was an indolopyridoquinazolinone alkaloid isolated from Evodia rutaecarpa and related herbs, which has shown a variety of intriguing biological properties. One important effect was anti-inflammatory [32]. It has been reported in the literature that PGRN can also play an anti-inflammatory role by blocking the inflammatory signaling pathway of TNFa-TNFR through antagonize TNFa [25]. The results suggested that the anti-inflammatory effect of Rutaecarpine may be achieved by promoting the expression of PGRN and suggested a potential application of the established reporter cell line in screening the small chemical drugs for therapy of the diseases involved in the inflammation and lipid metabolism. Above results indicated that the luciferase activity of the HEK293-PGRN-T2A-Luciferase-KI cell line was well correlated with the activity of the endogenous PGRN promoter.

In this study, a new luciferase knock-in reporter system was constructed that can accurately reflect the expression level of the endogenous PGRN gene. This system will be a useful tool for the functional study of PGRN and could have great potential in medical and industrial applications.
