# Supplementary material for: Generation of a novel HEK293 luciferase reporter cell line by CRISPR/Cas9-mediated site-specific integration in the genome to explore the transcriptional regulation of the PGRN gene
Source: Bioengineered. 2019 Apr 26;10(1):98–107. doi: 10.1080/21655979.2019.1607126 (PMC6527057; doi:10.1080/21655979.2019.1607126)
Supplement: Supplemental Material [file kbie-10-01-1607126-s001.docx]

Supplementary table 2**.** PCR primers used in the study.

| Primer | Sequence (5′→3′) | |
| --- | --- | --- |
| PGRN Knock-in DownArm nest forward | cacttctgccatgataaccaga | |
| PGRN Knock-in DownArm nest reverse | tccaccatggcgcagctcaa | |
| PGRN Knock-in DownArm *Sal*I forward | cgtcgacagaaagacatacacagccttaa | |
| PGRN Knock-in DownArm *Bgl*II reverse | cagatctacaactcgctgacgccgctgctggacga |  |
| PGRN Knock-in UpArm nest forward | cagtgggggatgtgaaatgtg | |
| PGRN Knock-in UpArm nest reverse | ggggtaatgtgatacagccga | |
| PGRN Knock-in UpArm *Cla*I forward | aatcgatatgggaggggacagcatctt | |
| PGRN Knock-in UpnArm *Xba*I reverse | ttctagacagcagctgtctcaaggctg | |
| PGRN Knock-in sgRNA detection forward | ctgcgagaaggaagtggtct | |
| PGRN Knock-in sgRNA detection reverse | ggggtaatgtgatacagccga | |
| PGRN Knock-in sgRNA detection nest forward | aggtgctgtaagcaggagag | |
| PGRN Knock-in sgRNA detection nest reverse | caaggacaacgtctaccgcaa | |
| PGRN Knock-in integration detection forward | cagtgggggatgtgaaatgtg | |
| PGRN Knock-in integration detection reverse | ggggtaatgtgatacagccga | |
| PGRN real time PCR forward | accgccagggcgtctgttgt | |
| PGRN real time PCR reverse | ggcctccctgcgcaaacact | |
