# Supplementary material for: Generation of a novel HEK293 luciferase reporter cell line by CRISPR/Cas9-mediated site-specific integration in the genome to explore the transcriptional regulation of the PGRN gene
Source: Bioengineered. 2019 Apr 26;10(1):98–107. doi: 10.1080/21655979.2019.1607126 (PMC6527057; doi:10.1080/21655979.2019.1607126)
Supplement: Supplemental Material [file kbie-10-01-1607126-s002.docx]

Supplementary Table 1. sgRNA [sequence](javascript:;)s used in the study

| Primer | Sequence (5′→3′) |
| --- | --- |
| PGRN Knock-in sgRNA1 forward | accgcagccctcgggaccccact |
| PGRN Knock-in sgRNA1 reverse | aaacagtggggtcccgagggctg |
| PGRN Knock-in sgRNA2 forward | accgcagagggcaccctccgagt |
| PGRN Knock-in sgRNA2 reverse | aaacactcggagggtgccctctg |
| PGRN Knock-in sgRNA3 forward | accgcagggaaggccttagattg |
| PGRN Knock-in sgRNA3 reverse | aaaccaatctaaggccttccctg |
| PGRN Knock-in sgRNA4 forward | accggccacagggtccactgaaa |
| PGRN Knock-in sgRNA4 reverse | aaactttcagtggaccctgtggc |
| PGRN promoter sgRNA1 forward | accgcgagggagtgttatgaatt |
| PGRN promoter sgRNA1 reverse | aaacaattcataacactccctcg |
| PGRN promoter sgRNA2 forward | accggcgcctgcaggatgggtta |
| PGRN promoter sgRNA2 reverse | aaactaacccatcctgcaggcgc |
| PGRN promoter sgRNA3 forward | accgcgtcgggacagcctcagca |
| PGRN promoter sgRNA3 reverse | aaactgctgaggctgtcccgacg |
| PGRN promoter sgRNA4 forward | accgttggagaatcatgtgacgt |
| PGRN promoter sgRNA4 reverse | aaacacgtcacatgattctccaa |
| PGRN promoter sgRNA5 forward | accgatctctagcaaactccccc |
| PGRN promoter sgRNA5 reverse | aaacgggggagtttgctagagat |
| PGRN promoter sgRNA6 forward | accggagatgatagcgcgtgtct |
| PGRN promoter sgRNA6 reverse | aaacagacacgcgctatcatctc |
| PGRN promoter sgRNA7 forward | accgtggggcattgacagtgcga |
| PGRN promoter sgRNA7 reverse | aaactcgcactgtcaatgcccca |
